# Comparison of Energy Contributions and Workloads in Male and Female Badminton Players During Games Versus Repetitive Practices

**DOI:** 10.3389/fphys.2021.640199

**Published:** 2021-06-24

**Authors:** Yue Fu, Yu Liu, Xiaoping Chen, Yongming Li, Bo Li, Xinxin Wang, Yang Shu, Lei Shang

**Affiliations:** ^1^School of Kinesiology, Shanghai University of Sport, Shanghai, China; ^2^Key Laboratory of Exercise and Health Sciences of Ministry of Education, Shanghai University of Sport, Shanghai, China; ^3^China Institute of Sport Science, Beijing, China; ^4^School of Physical Education and Sport Training, Shanghai University of Sport, Shanghai, China; ^5^School of Competitive Sport, Beijing Sport University, Beijing, China

**Keywords:** athlete monitoring, match loads, smash training, energy supply, triaxial accelerometer

## Abstract

**Purpose:**

The aim of this study was to compare the energy contributions and workloads in men and women during badminton matches versus frequently used multi-ball smash practices.

**Methods:**

Fourteen badminton players performed one badminton singles game and one session of smashing practice on separate days. The energy contributions were examined in terms of each individual’s three energy systems and substrate oxidation, while workloads included heart rate (HR), Player Load (PL), accelerations, decelerations, changes of direction, and jumps.

**Results:**

(1) During games, male players exhibited higher adenosine triphosphate–phosphocreatine system contribution (E_PCr_, kJ) (*p* = 0.008) and average rate of carbohydrate oxidation (R_CHO_, g/min) (*p* = 0.044) than female players, while female players showed greater absolute PL (*p* = 0.029) and more accelerations (*p* = 0.005) than male players. Furthermore, players who lost performed higher relative PL (*p* = 0.017) than those who won. (2) Higher energy system contributions, including E_PCr_ (kJ) (*p* = 0.028), E_HLa_ (kJ) (*p* = 0.024), E_Aer_ (kJ) (*p* = 0.012), E_Tot_ (kJ) (*p* = 0.007), and R_CHO_ (g/min) (*p* = 0.0002), were seen in male players during repetitive spike practices. Male players also made greater number of jumps (*p* = 0.0002). (3) Players exhibited higher aerobic energy contribution (*p <* 0.001), mean HR (*p* = 0.002), and HRmax (*p* = 0.029) during games, while exhibiting greater anaerobic energy contribution (*p <* 0.001) and relative PL (*p* = 0.001) during repetitive practices.

**Conclusion:**

The similarities between male and female badminton players in proportional use of the three energy systems during games and repetitive spike training indicate similar relative energy demands for both genders. However, considering the need for higher aerobic capacity in competition, it might be advisable to design appropriate work:rest ratios for repetitive practices in daily training.

## Introduction

Badminton is a physically demanding racquet sport that involves frequent bouts of high-intensity activity, and a complex skill concerning repeated acceleration, deceleration, changes of direction (CoD), and jumps (Jum) (Cabello and Gonzalez, 2003; Abdullahi et al., 2019). Well-trained badminton players are able to stroke using a diverse set of sport-specific techniques at varying frequencies throughout a match. To improve various kinds of stroke techniques, high-repetition practices are used extensively during daily training. However, the workload of high-repetition stroke techniques has received limited attention. A deeper understanding of the high-repetition techniques may help to develop sport-specific training programs that could enhance performance in competitive badminton. The contribution of each energy system in matches and for specific badminton skills is unclear. Previous studies exploring the energetic profile of badminton players in games showed a 60–70% aerobic-dominant profile (Chin et al., 1995; Faccini and Dai Monte, 1996; Deka et al., 2017), and some scientists found the adenosine triphosphate–phosphocreatine (ATP-PCr) system (E_PCr_) and the glycolysis system (E_HLa_) to be the main suppliers of energy (Li and Ling, 1997). However, energy contributions may be influenced by different physical loads, such as different strokes, foot movements and the frequencies with which these actions take place. Therefore, a better understanding of the badminton player’s energy contribution can only be established by investigating the energetic profile for each combination of these and similar actions.

Quantifying the physiological and physical loads imposed by competitions and training drills is vital to understanding the dose–response nature of the exercise process with regard to optimizing players’ performances. Athletic ability, gender, and posture are related to badminton injuries by understanding loading characteristics. For example, unskilled female players have been shown to be more vulnerable to lower extremity injuries (Lam et al., 2018), and postures have been associated with knee injuries during badminton games (Sasaki et al., 2018).

Workloads have been extensively investigated in different sports (Garcia et al., 2019, 2020; McFadden et al., 2020). Workloads include heart rate (HR), rating of perceived exertion (RPE), Player Load (PL), accelerations (Acc), decelerations (Dec), CoD, Jum, and so on. Although studies that quantify these loads are mostly limited to match performance or selected training periods (Bartlett et al., 2017; Simpson et al., 2020; Taylor et al., 2020), the loads required in various sport-specific practices are equally important. Liu found that the player’s lower back is an ideal location for a wearable sensor capable of monitoring overall badminton external loads (Liu et al., 2021). Trivial to moderate relationships have been found between internal and external match loads in male, singles badminton players (Abdullahi et al., 2019). However, additional research comparing energy contributions and workloads in male and female badminton players is warranted in order to determine potential gender differences in practice strategies and recovery needs. At present, no study has compared the energy contributions and workloads in men and women during badminton matches and repetitive training.

Therefore, the main purpose of this study was to compare the energy contributions and workloads in male and female badminton players. The further aim was to describe differences of energy contributions and workloads between badminton matches and intermittent stroke practices with a 1:2 work:rest ratio.

## Materials and Methods

### Participants

Fourteen healthy sub-elite badminton players who competed at the national level in their age group volunteered to participate in this study. The players stopped training 24 h before testing. They were instructed to maintain a regular diet and not to perform additional vigorous exercise during the experiment. On the day of testing, participants finished breakfast at least 1 h before reporting to the training center. All participants were medically screened to ensure no contraindications to study participation. Anthropometric and performance characteristics of these participants are presented in Table 1. Prior to the study, the players, their coaches, and guardians were informed of the test procedures and potential risks. After having the benefits and risks explained to them, the players and their guardians provided informed written consent. Ethical approval (approval number: 20200901) was obtained from the research ethics committee of the China Institute of Sport Science, Beijing, China.

**TABLE 1:** Participants’ characteristics.

	**Age (years)**	**Height (cm)**	**Body mass (kg)**	**Training experience (years)**
Males (*N* = 8)	18.25 ± 3.41	181.88 ± 9.26	70.99 ± 17.80	10.88 ± 3.00
Females (*N* = 6)	16.50 ± 2.51	168.67 ± 3.88	54.95 ± 5.86	9.17 ± 2.56
Total (*N* = 14)	17.50 ± 3.08	176.21 ± 9.90	64.12 ± 15.87	10.14 ± 2.85

*Data are presented as mean ± SD.*

### Design and Procedures

The study design was cross-sectional. All participants performed one badminton singles game and one session of repetitive spike practice on separate days (both tests were conducted indoors at similar times of day: players who performed in the morning or afternoon also practiced in the morning or afternoon). Energy contributions and workloads were monitored by simultaneous gas exchange measurements, HR technology and accelerometer technology during games and practices. Before the formal test, the players performed 15 min sparring practice and dynamic stretching to warm up. After sitting still for 10 min, they put on the portable spirometry system (K4b^2^, Cosmed, Rome, Italy), HR monitor (Polar Accurex Plus, Polar Electro Inc., Kempele, Finland), and Catapult OptimEye S5 (Catapult Sports, Melbourne, VIC, Australia). Standard calibration was performed with a 3-L syringe and a standard gas with a known composition (O_2_: 15.00%, CO_2_: 5.09%), which was corrected for barometric pressure and humidity prior to the test. Players wore the abovementioned devices during the games and practice sessions. Prior to the warmup, immediately before the first set, between each set and during the recovery period following the games or practice, 10 μl of capillary blood was collected from the ear lobe to determine the blood lactate concentration (Biosen C line, EKF Diagnostic, Magdeburg, Germany). The accumulated blood lactate values were used to calculate the energy from the anaerobic lactic pathway.

#### Badminton Match Play

**FIGURE 1:**
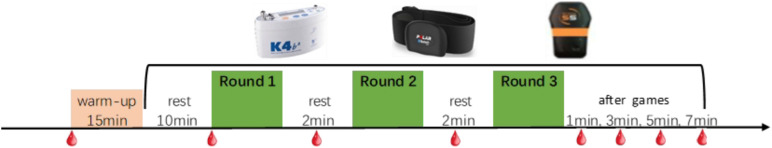
Test flow chart of a badminton match.

Prior to the formal test, the players were divided into seven pairs for the singles games; pairing was organized to ensure that players were of a similar skill level. They were also instructed to dress and eat as they usually would for a match. The games would follow the rules of the International Badminton Federation: three games played to 21 points; if both players score 20 points, a player must lead his or her opponent by 2 points to win. During the game, when the leading player scored 11 points, both players took a 1-min rest. Between the rounds, players had a 2-min break. The competitions were judged by a national referee. In order to make the test resemble an actual game as much as possible, the winners and losers were given different rewards as incentives. The specific test process is shown in Figure 1.

### Repetitive Stroke Practice

**FIGURE 2:**
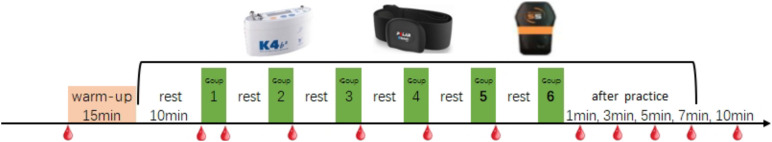
Test flow chart of multi-ball stroke practice for each player.

Fourteen players performed six sets of spike practice 10 times. An experienced coach was responsible for continuous serves from the other half of the court to ensure that the participants could perform the overhead stroke smoothly. After each set, the players took a break (sitting still) for twice the exercise time. The specific test process is shown in Figure 2.

#### Energy Contributions Monitoring

The energy contributions monitored by portable spirometry included the ATP-PCr system/anaerobic alactic contribution (E_PCr_), the glycolytic system/anaerobic lactic energy contribution (E_HLa_), the oxidative system/aerobic energy contribution (E_Aer_), the total energy contribution (E_Tot_), the average rate of carbohydrate oxidation (R_CHO_), and the average rate of lipid oxidation (R_Lip_). Calculations of the average energy contributions and energy costs were made using gas exchange data that were recorded during the test and rest periods.

##### Calculation of the energy system’s contributions

To estimate the energy expenditure of all tests, the sum of the contributions of the three energy systems was determined in accordance with the methodology used by other studies in sports (di Prampero, 1981; Davis et al., 2014; Julio et al., 2017; Li et al., 2018, 2020):

(1) The ATP-PCr system contribution was shown as E_PCr_ and estimated using the first 3-min fast phase of the $\dot{V}$O_2_ after exercise (games or practices), with a caloric equivalent of 0.021131 kJ/ml at respiratory exchange ratio >1.0. The first 3-min slow phase of the $\dot{V}$O_2_ was determined by using an approximated exponential equation estimated from a non-linear fitting procedure. The equation was derived from the actual $\dot{V}$O_2_ of the second 3 min after exercise.

FastphaseoftheV.O(ml)2

=actualV.O(ml)2-slowphaseoftheV.O(ml)2

E(kJ)PCr=fastphaseoftheV.O(ml)2×0.021131(kJ/ml)

(2) The glycolytic system contribution, shown as anaerobic lactic energy contribution (E_HLa_), was calculated from the accumulated blood lactate during the test (maximal value subtracted resting value) with the O_2_-lactate equivalent of 3.0 ml/mM/kg (assuming that the accumulation of 1 mM in lactate was equivalent to 3 ml O_2_ per kilogram of body mass). Resting blood lactate was the value before warmup and maximal blood lactate was the largest of all values.

Accumulated⁢blood⁢lactate⁢(mM)=maximal⁢blood⁢lactate⁢(mM)-resting⁢blood⁢lactate⁢(mM)

E(kJ)HLa=accumulatedbloodlactate(mM)×3.0⁢(ml/mM/kg)×body⁢mass⁢(kg)×0.021131⁢(kJ/ml)

(3) The oxidative system contribution, shown as aerobic energy contribution (E_*A*__*er*_), was calculated from the accumulated $\dot{V}$O_2_ during test above resting levels, with a caloric equivalent of 0.021131 kJ/ml at respiratory exchange ratio >1.0. Rest levels were defined as 4.0 ml/min/kg for males and 3.5 ml/min/kg for females in a standing posture. Total $\dot{V}$O_2_ during exercises (games or practices) was calculated from the portable spirometry system and expressed in milliliters.

Resting⁢V.⁢O⁢for2⁢males⁢(ml)=4.0⁢(ml/min/kg)×bodymass⁢(kg)×duration⁢(min)

Resting⁢V.⁢O⁢for2⁢females⁢(ml)=3.5⁢(ml/min/kg)×bodymass⁢(kg)×duration⁢(min)

AccumulatedV.O(ml)2=totalV.O(ml)2-restingV.O(ml)2

E(kJ)Aer=accumulatedV.O(ml)2×0.021131(kJ/ml)

(4) The total energy expenditure (E_T__ol_) was computed as the sum of E_PCr_, E_HLa_, and E_*A*__*er*_. In addition, the contributions of the three energy systems were expressed as a percenage of the total energy expenditure.

E(kJ)Tot=E(kJ)PCr+E(kJ)HLa+E(kJ)Aer

E(%)PCr=E(kJ)PCr÷E(kJ)Tot×100%

E(%)HLa=E(kJ)HLa÷E(kJ)Tot×100%

E(%)Aer=E(kJ)Aer÷E(kJ)Tot×100%

##### Calculation of substrate oxidation

Substrate oxidation was estimated for the interval session, including work and rest periods. Carbohydrate and lipid oxidation rates were calculated by the non-protein respiratory quotient (Peronnet and Massicotte, 1991; Pettersson et al., 2019), Oxygen consumption ($\dot{V}$O_2_) and carbon dioxide production ($\dot{V}$CO_2_) were expressed in liters per minute (L/min) and oxidation rate in grams per minute (g/min):

Carbohydrate⁢oxidation⁢rate=(4.585×V.CO)2-(3.226×V.O)2

Lipid⁢oxidation⁢rate=(1.695×V.O)2-(1.701×V.CO)2

#### Workloads Monitoring

The workload variables used in this study were HR, absolute PL, relative PL, Acc, Dec, CoD (left and right), and Jum. A 10-Hz GPS device fitted with a 100-Hz triaxial accelerometer, gyroscope, and magnetometer (OptimEye S5, Catapult Sports, Melbourne, VIC, Australia) was securely positioned between the participant’s scapulae using a custom-made vest. The device firmware version was 7.40. The data were processed by the manufacturer’s software (OpenField, v1.21.1, Catapult Sports, Melbourne, VIC, Australia). The numbers of Acc, Dec, CoD, and Jum and CoD, including both left turns (CoD left) and right turns (CoD right), were measured by the device’s inertial sensors, throughout the test. (Data between sets were excluded). Absolute PL was defined as the sum of the acceleration vectors as assessed through the accelerometer (Catapult OptimEye S5) in three axes (lateral, vertical, and anterior/posterior). Relative PL was determined as PL per minute in each period. Both absolute PL and relative PL were measured in arbitrary units (au). The PL variable demonstrated strong validity and reliability indices to assess the neuromuscular load of each referee, and the corresponding value was calculated through the following equation:

$$\mathrm{Player}\,{\mathrm{Load}}_{t=n}=\sum_{t=0}^{t=n}\sqrt{\frac{{\left(X_{t=n}-X_{t=n-1}\right)}^{2}\,{\left(Y_{t=n}-Y_{t=n-1}\right)}^{2}\,{\left(Z_{t=n}-Z_{t=n-1}\right)}^{2}}{100}}$$

where *X* refers to acceleration in the medial–lateral direction, *Y* refers to vertical acceleration, and *Z* represents acceleration from the anterior-to-posterior direction. Time is represented by *t* and *n* refers to number.

### Statistical Analyses

Statistical analyses were conducted using the IBM SPSS statistical software (version 25.0, IBM Corporation, Armonk, NY, United States). Results were expressed as means ± standard deviations (SD). The Shapiro–Wilk test was used to assess normality. Comparisons between male and female players as well as players who won their matches compared with the losing players were carried out using independent-sample *t*-tests if data satisfied normal distribution. Otherwise, non-parametric test (Mann–Whitney *U* test) was used to compare groups. Significance level was set at *p* < 0.05.

## Results

### Results of Energy Contributions and Workloads During Game

The 14 players played a total of seven games. Six matches reached two rounds; only one game (female) had three rounds. The average match duration was 24.93 ± 6.33 min. Statistics for the energy contributions and workloads are presented in Table 2. According to non-parametric distributions, only the comparisons of absolute PL (AU) and decelerations (*n*) between men and women and the comparisons of E_PCr_ (%), relative PL (AU), and jumps (*n*) between victory and defeat used an on-parametric test (Mann–Whitney *U* test); other variables used independent-sample *t*-tests. There were statistically significant differences in E_PCr_ (kJ), R_CHO_(g/min), absolute PL, and accelerations (*n*) between male and female players (*p* < 0.05). Male players exhibited higher anaerobic alactic capacity (*p* = 0.008) and average rate of carbohydrate oxidation (*p* = 0.044) than female players. The female players showed greater workloads in absolute PL (*p* = 0.029) and number of accelerations (*p* = 0.005) compared with their male counterparts. No other significant gender differences were seen during a match. Furthermore, defeated players exhibited a higher relative PL than winners (*p* = 0.017).

**TABLE 2:** Descriptive results of energy contributions and workloads during a badminton match.

		**Males**	**Females**	**Victory**	**Defeat**	**Total**
Energy contributions	E_PCr_ (kJ)	45.04 ± 10.04	28.55 ± 5.14*	44.82 ± 13.43	33.42 ± 8.19	38.17 ± 11.69
	E_HLa_ (kJ)	13.62 ± 11.04	7.30 ± 3.19	12.96 ± 10.84	9.67 ± 8.11	11.19 ± 9.20
	E_Aer_ (kJ)	832.07 ± 175.63	826.76 ± 226.96	817.37 ± 213.89	840.88 ± 178.66	830.03 ± 187.54
	E_Tot_ (kJ)	920.82 ± 164.26	862.62 ± 224.83	914.19 ± 214.84	883.98 ± 176.39	896.57 ± 184.38
	E_PCr_ (%)	5.01 ± 1.47	3.51 ± 1.09	5.05 ± 1.83	3.90 ± 1.09	4.38 ± 1.48
	E_HLa_ (%)	1.74 ± 1.61	0.87 ± 0.42	1.74 ± 1.74	1.11 ± 0.93	1.38 ± 1.29
	E_Aer_ (%)	93.25 ± 2.98	95.62 ± 1.38	93.20 ± 3.52	94.98 ± 1.75	94.24 ± 2.65
	R_CHO_ (g/min)	1.56 ± 0.69	0.96 ± 0.12*	1.52 ± 0.81	1.17 ± 0.38	1.33 ± 0.62
	R_Lip_ (g/min)	0.67 ± 0.16	0.52 ± 0.08	0.64 ± 0.19	0.59 ± 0.12	0.61 ± 0.15
Workloads	Mean HR (bpm)	162.38 ± 18.35	171.17 ± 8.93	168.57 ± 19.42	163.71 ± 10.63	166.14 ± 15.25
	HRmax (bpm)	194.50 ± 15.00	198.50 ± 4.76	194.29 ± 13.61	198.14 ± 9.84	196.21 ± 11.58
	Absolute PL (AU)	111.85 ± 19.77	147.82 ± 31.24*	118.76 ± 24.49	135.77 ± 35.21	127.27 ± 30.44
	Relative PL (AU)	5.02 ± 0.26	4.92 ± 0.86	4.66 ± 0.39	5.29 ± 0.57^Δ^	4.98 ± 0.57
	Accelerations (*n*)	41.75 ± 14.92	72.50 ± 18.63*	50.00 ± 17.23	59.86 ± 27.12	54.93 ± 22.42
	Decelerations (*n*)	46.38 ± 29.91	68.17 ± 29.86	55.71 ± 35.68	55.71 ± 28.02	55.71 ± 30.82
	CoD left (*n*)	144.13 ± 40.76	165.67 ± 75.82	143.00 ± 62.66	163.71 ± 53.08	153.36 ± 56.81
	CoD right (*n*)	82.25 ± 35.25	70.00 ± 11.54	83.86 ± 37.50	70.14 ± 11.39	77.00 ± 27.56
	Jumps (*n*)	27.63 ± 16.17	31.50 ± 19.58	30.71 ± 20.69	27.86 ± 14.16	29.29 ± 17.09

Values are expressed as means ± SD. E_PCr_, ATP-PCr system contribution; E_HLa_, glycolytic system contribution; E_Aer_, aerobic energy contribution; E_Tot_, total energy contribution; R_CHO_, average rate of carbohydrate oxidation; R_Lip_, average rate of lipid oxidation; HR, heart rate; Max, maximum; PL, Player Load; CoD, changes of direction. *Significantly different from males (*p* < 0.05).

*^Δ^Significantly different from victory (*p* < 0.05).*

### Results of Energy Contributions and Workloads During Repetitive Stroke Practice

The average spike training session was 11.4 ± 0.45 min. No significant difference in duration was seen between male and female players. Descriptive statistics of energy contributions and training load-related results are presented in Table 3. Only comparisons of E_PCr_ (%), accelerations (*n*), decelerations (*n*), CoD Left (*n*), and CoD Right (*n*) between men and women used an on-parametric test (Mann–Whitney *U* test), because these data exhibited non-parametric distributions, and other variables used independent-sample *t*-tests. Higher energy system contributions, including E_PCr_ (kJ) (*p* = 0.028), E_HLa_ (kJ) (*p* = 0.024), E_Aer_ (kJ) (*p* = 0.012), E_Tot_ (kJ) (*p* = 0.007), and R_CHO_ (g/min) (*p* = 0.0002), were seen in male players; there was no difference in the percentages of the three energy systems. Male players accumulated a significantly greater number of jumps (*p* = 0.0002) during multi-ball spike practices, but no differences in other training load variables were seen between genders.

**TABLE 3:** Descriptive results of energy contributions and workloads during stroke practices.

		**Males**	**Females**	**Total**
Energy contributions	E_PCr_ (kJ)	43.37 ± 5.53	32.07 ± 11.17*	38.53 ± 9.91
	E_HLa_ (kJ)	48.32 ± 24.81	19.86 ± 12.09*	36.12 ± 24.52
	E_Aer_ (kJ)	439.16 ± 88.83	325.46 ± 32.61*	390.43 ± 89.82
	E_Tot_ (kJ)	530.85 ± 108.10	377.39 ± 45.99*	465.09 ± 115.40
	E_PCr_ (%)	8.55 ± 2.61	8.34 ± 2.31	8.46 ± 2.39
	E_HLa_ (%)	8.71 ± 3.15	5.18 ± 3.04	7.20 ± 3.49
	E_Aer_ (%)	82.74 ± 3.31	86.48 ± 3.38	84.34 ± 3.74
	R_CHO_ (g/min)	2.44 ± 0.58	1.10 ± 0.26*	1.86 ± 0.82
	R_Lip_ (g/min)	0.56 ± 0.10	0.51 ± 0.11	0.54 ± 0.10
Workloads	Mean HR (bpm)	146.57 ± 10.42	149.73 ± 14.44	147.92 ± 11.89
	HRmax (bpm)	189.38 ± 8.00	185.50 ± 6.53	187.71 ± 7.41
	Absolute PL (AU)	77.78 ± 6.78	68.71 ± 14.10	73.89 ± 11.09
	Relative PL (AU)	6.30 ± 0.63	5.60 ± 0.91	6.00 ± 0.81
	Accelerations (*n*)	3.38 ± 2.62	7.83 ± 10.01	5.29 ± 6.89
	Decelerations (*n*)	4.38 ± 4.03	3.17 ± 3.82	3.86 ± 3.84
	CoD left (*n*)	37.88 ± 11.78	31.50 ± 12.55	35.14 ± 12.08
	CoD right (*n*)	16.50 ± 17.91	4.83 ± 3.54	11.50 ± 14.61
	Jumps (*n*)	61.25 ± 21.71	9.17 ± 10.61*	38.93 ± 31.82

Values are expressed as means ± SD. E_PCr_, ATP-PCr system contribution; E_HLa_, glycolytic system contribution; E_Aer_, aerobic energy contribution; E_Tot_, total energy contribution; R_CHO_, average rate of carbohydrate oxidation; R_Lip_, average rate of lipid oxidation; HR, heart rate; Max, maximum; PL, Player Load; CoD, changes of direction.

*Significantly different from males (*p* < 0.05).

### Differences in Energy Contributions and Workloads Between Game and Repetitive Stroke Practice

Comparisons of energy contributions and workloads in single-player games and multi-ball spike training are presented in Table 4. Only some indicators (those less influenced by duration) were selected. Since data exhibited non-parametric distributions, comparisons of E_HLa_ (%), E_Aer_ (%), and R_CHO_ (g/min) between games and stroke practices used an on-parametric test (Mann–Whitney *U* test); other variables used independent-sample *t*-tests. Among these indicators, players exhibited higher E_PCr_ (%) (*p* = 0.00003) and E_HLa_ (%) (*p <* 0.001) during repetitive spike training, and higher aerobic energy contribution (*p <* 0.001) during games. Higher mean HR (*p* = 0.002) and max HR (*p* = 0.029) were found during games, but greater relative PL (*p* = 0.001) was seen in multi-ball spike training.

**TABLE 4:** Differences of energy contributions and workloads between games and practices.

		**Game**	**Practice**
Energy contributions	E_PCr_ (%)	4.38 ± 1.48	8.46 ± 2.39*
	E_HLa_ (%)	1.38 ± 1.29	7.20 ± 3.49*
	E_Aer_ (%)	94.24 ± 2.65	84.34 ± 3.74*
	R_CHO_ (g/min)	1.33 ± 0.62	1.86 ± 0.82
	R_Lip_ (g/min)	0.61 ± 0.15	0.54 ± 0.1
Workloads	Mean HR (bpm)	166.14 ± 15.25	147.92 ± 11.89*
	HRmax (bpm)	196.21 ± 11.58	187.71 ± 7.41*
	Relative Player Load (AU)	4.98 ± 0.57	6.00 ± 0.81*

Values are expressed as means ± SD. E_PCr_, ATP-PCr system contribution; E_HLa_, glycolytic system contribution; E_Aer_, aerobic energy contribution; E_Tot_, total energy contribution; R_CHO_, average rate of carbohydrate oxidation; R_Lip_, average rate of lipid oxidation; HR, heart rate; Max, maximum; PL, Player Load; CoD, changes of direction.

*Significant differences between game and multi-ball spike training.

## Discussion

Badminton matches last around 28–78 min (10–21 min/round) and are fast paced with intermittent moments. The duration of a single bout is about 6–12 s, and the number of shots in a bout is around 5–12 strokes (Faude et al., 2007; Abián-Vicén et al., 2013; Abian et al., 2014; Gawin et al., 2015; Laffaye et al., 2015; Kah Loon and Krasilshchikov, 2016; Savarirajan, 2016). These bouts involve multiple accelerations, decelerations, CoD, and jumps, which can raise one’s HR to 95% of its maximum level (HRmax) (Gawin et al., 2015; Laffaye et al., 2015; Abdullahi and Coetzee, 2017). However, the workloads and average intensity are not very high over the entire duration of a match due to the occurrence of low-intensity intervals between bouts, which is characterized by 72.6–74.8% $\dot{}\,\dot{\text{V}}$O_2_max, 70–85% HRmax, and 1.98–4.6 mM blood lactate concentration (Majumdar et al., 1997; Cabello and Gonzalez, 2003; Faude et al., 2007; Sung, 2016). Other studies have revealed that the energy consumption is significantly greater in singles matches when compared with doubles matches and that these differences are not related to a player’s gender (Lee, 2013). Similar to the findings of the present study, gender differences in activity patterns induced only slightly different physiological responses (Fernandez et al., 2013). Our investigation explored the energy contributions and workloads in male and female badminton players: while the similarities in proportional use of the three energy systems between male and female badminton players during games and training sessions indicate similar relative energy demands for both genders, male players showed higher E_PCr_ (kJ) during games and greater energy contributions, including E_PCr_ (kJ), E_HLa_ (kJ), E_Aer_ (kJ), and E_Tot_ (kJ), during spike practices than female players. At the same time, male players had a higher average rate of carbohydrate oxidation during games and repetitive practice sets. This suggests that players (especially males) should enhance carbohydrate supplementation during competition and high-intensity training. Additionally, female players showed greater workloads in absolute PL and the number of accelerations compared with male players, while male players accumulated a significantly greater number of jumps during spike practices. No other differences in workload variables between genders were observed. In contract to the present study, Rojas-Valverde et al. (2020) found gender-related differences in maximum accelerations, relative accelerations, and relative distance during games. This is most likely related to the monitoring equipment’s method of generating statistics for jumping: the equipment used in this study only recorded jumps when both feet were off the ground simultaneously at a certain vertical height; it did not record as jumps those movements in which only one foot left the ground or when one foot left the ground, then the other. The different jumping styles and heights between genders likely explain the difference in the number of jumps recorded. Differences in workloads between this study and other studies may be related to the dissimilarities in type, intensity, and duration of the activities involved (Ghosh et al., 1990, 1993; Chin et al., 1995; Faude et al., 2007; Aydogmus, 2015; Deka et al., 2017). For instance, the frequency and movement pattern during an overhead stroke may differ between players (Sasaki et al., 2020) while lunging during underhand strokes on the dominant hand side leg had greater mediolateral acceleration than other movements (Nagano et al., 2020).

The importance of the aerobic energy supply in badminton was underestimated in some studies, which observed that 60–70% energy is contributed by the aerobic system and approximately 30% by the anaerobic system, with greater demand on the anaerobic alactic metabolism than the lactic anaerobic metabolism (Phomsoupha and Laffaye, 2015). The results of this study, which observed that almost 95% energy is contributed by the aerobic system, pointed to the need for a higher aerobic capacity in competitive badminton players. Daily training should be designed to further develop a sufficient endurance capacity.

Furthermore, there are great differences in the proportions of the three energy systems between competition and the intermittent spike training with a 1:2 work:rest ratio. Players exhibited higher aerobic energy contribution during games, and higher anaerobic energy contribution during spike training. This suggests that we should put particular emphasis on the aerobic ability of badminton players and that a larger intermittent work:rest ratio of each repetitive drill should be considered. Integrated training programs should be conducted to combine physical demands with decision-making demands. Aerobic assessment using indirect calorimetry is impractical on the court due to the burden of wearing portable metabolic devices. Some coaches use $\dot{\text{V}}$O_2_max to distinguish players’ levels, but Ooi observed that $\dot{\text{V}}$O_2_max may not discriminate elite badminton players from sub-elite counterparts, suggesting that tactical knowledge and psychological readiness could be more important for elite athletes (Ooi et al., 2009). We suggest that players at different levels of expertise should undertake different training regimens, with different work:rest ratios and overall durations. Given that HR monitoring may not provide accurate data on the energetic demands for badminton players, an indirect calorimetry test on court to assess energetic demands would be more precise (Rampichini et al., 2018). Nevertheless, the results from laboratory treadmill testing seem to be a poor predictor of a player’s ability, compared to their game play performance (Heller, 2010).

The present study’s limitations include a lack of repeated match and training data and the relatively small sample size. Future research into both energy contributions and workloads derived from laboratory experiments is warranted in order to understand the relative differences in workloads of each player. The need for additional research also applies to the determination of sprint classifications specific to badminton, as well as game-specific CoD or acceleration.

Consequently, we encourage measuring these activities both in future research and during practices throughout the sports season. Future studies should expand our knowledge of energy contributions and workloads in routine badminton drills, including spike training with different work:rest ratios and other combinations of badminton techniques. Additionally, this study should be made of badminton players at different levels of expertise.

## Applications and Conclusion

Our findings highlight the similarities in proportional use of the three energy systems between male and female badminton players throughout competition and repetitive spike training. Players (especially males) should enhance carbohydrate supplementation during competition and high-intensity training in accordance with the higher carbohydrate oxidation rate observed.

Study results suggest that there are important differences in the contributions of the three energy systems between competition and repetitive spike training. Considering the need for higher aerobic capacity in competition, it may be practical for badminton coaches and athletes to choose appropriate intermittent work:rest ratio in this technique during high-repetition practices. We suggest that players at different competitive levels should undertake training regimens of different work:rest ratios and overall durations.

Monitoring and quantifying energy contributions and workloads during matches and training are indispensable for determining individualized training regimes. Training programs should be adjusted according to specific competitive characteristics in accordance with the demands of different sports. Wearable technologies are an efficient method for monitoring workloads throughout the season in order to help enhance players’ performances.

## Data Availability Statement

The raw data supporting the conclusions of this article will be made available by the authors, without undue reservation.

## Ethics Statement

The studies involving human participants were reviewed and approved by the Ethics Committee of China Institute of Sport Science, China. Written informed consent to participate in this study was provided by the participants’ legal guardian/next of kin.

## Author Contributions

YL and XC contributed to conception and design of the study. YL, BL, and XC designed the study. XW and LS collected the data. XW and YF conducted the analyses. YF and YS wrote the manuscript. All authors read and approved the final version of the manuscript.

## Conflict of Interest

The authors declare that the research was conducted in the absence of any commercial or financial relationships that could be construed as a potential conflict of interest.
